# Sequence differences at orthologous microsatellites inflate estimates of human-chimpanzee differentiation

**DOI:** 10.1186/1471-2164-15-990

**Published:** 2014-11-18

**Authors:** Michelle Kwong, Trevor J Pemberton

**Affiliations:** Department of Biochemistry and Medical Genetics, University of Manitoba, Winnipeg, Manitoba Canada

## Abstract

**Background:**

Microsatellites---contiguous arrays of 2–6 base-pair motifs---have formed the cornerstone of population-genetic studies for over two decades. Their genotype data typically takes the form of PCR fragment lengths obtained using locus-specific primer pairs to amplify the genomic region encompassing the microsatellite. Recently, we reported a dataset of 5,795 human and 84 chimpanzee individuals with genotypes at 246 human-derived autosomal microsatellites as a resource to facilitate interspecies comparisons. A major assumption underlying this dataset is that PCR amplicons at orthologous microsatellites are commensurable between species.

**Results:**

We find this assumption to be frequently incorrect owing to discordance in microsatellite organization and variability, as well as nontrivial length imbalances caused by small species-specific indels in microsatellite flanking sequences. Converting PCR fragment lengths into the repeat numbers they represent at 138 microsatellites whose organization and variability was found to be highly similar in both species, we show that interspecies incommensurability among PCR amplicons can inflate *F*_ST_ and *D*_PS_ estimates by up to 10.6%. Separate investigations of determinants of microsatellite variability in humans and chimpanzees uncover similar patterns with mean and maximum numbers of repeats, as well as numbers and ranges of distinct alleles, all important factors in predicting heterozygosity. In contrast, across microsatellites, numbers of repeats were significantly smaller in chimpanzees than in humans, while numbers and ranges of distinct alleles were instead larger.

**Conclusions:**

Our findings have fundamental implications for interspecies comparisons using microsatellites and offer new opportunities for more accurate comparisons of patterns of human and chimpanzee genetic variation in numerous areas of application.

**Electronic supplementary material:**

The online version of this article (doi:10.1186/1471-2164-15-990) contains supplementary material, which is available to authorized users.

## Background

Understanding human evolutionary history in the context of the colonization of the major continental regions by anatomically modern humans (AMH) over the past ~60-125,000 years [1–3] has been a major focus of human population genetics since Charles Darwin and Thomas Huxley first proposed a single African origin for AMH and a shared ancestry between humans and chimpanzees [4, 5]. Molecular studies over the past several decades have subsequently confirmed recent common ancestry between humans and the great apes [6–9] with common chimpanzees (*Pan troglodytes*) and bonobos (*Pan paniscus*) our closest living relatives. In addition, studies of neutral genetic variation in the mitochondrial genome [10–14] and on the Y-chromosome [15–17] have provided strong support for a common African origin of AMH [18]. Furthermore, analyses of autosomal genetic variation in the form of microsatellites [19, 20] uncovered patterns consistent with a serial migration of AMH outward from Central Africa [21–24] as well as a time to the most recent common ancestor between humans and chimpanzees of 5.8-9.8 million years ago [25].

Microsatellites consist of short arrays of tandemly reoccurring repeats (STR) of a 2–6 bp motif that vary in length between individuals and that generally have many distinct alleles within a population. Abundant in diverse genomes [26–30], they are among the fastest-evolving DNA sequences with relatively high mutation rates of at least 10^-4^–10^-3^ events per microsatellite per gamete per generation in humans [25, 31–37] and other mammals [38–40]. Microsatellites mutate via a slipped-strand mispairing process during DNA replication [41–45] and broadly follow a stepwise mutation model [46], with ~68% of mutations at microsatellites with a dinucleotide repeat unit and >96% of mutations at microsatellites with a tetranucleotide repeat unit involving a change of a single repeat [25, 35]. It is their high level of mutability compared to other genomic regions [31, 47–50] and stepwise relationship between alleles that afford multiallelic microsatellites their generally higher informativeness in genetic studies than less variable markers such as biallelic single nucleotide polymorphisms (SNPs) [51–54].

Since the landmark paper by Bowcock *et al*. [55] demonstrated the utility of microsatellites for the investigation of human evolutionary genetics, they have been used extensively to investigate genetic variation patterns among worldwide human populations. Subsequent population-genetic studies have frequently utilized standardized genome-wide panels originally designed for linkage analysis [56] that comprise hundreds of microsatellites genotyped in hundreds to thousands of individuals [19, 20, 57–64]. In addition, an investigation into genetic variation patterns among chimpanzees utilized genotype data at putative orthologs of 310 human-derived microsatellites that overlapped those used in human studies [65]. Recently, we reported the largest microsatellite dataset of its kind to date that subsumed these human and chimpanzee datasets and comprised 246 autosomal microsatellites common to all studies with genotypes in 5,795 individuals from 267 human populations and in 84 individuals from six chimpanzee groups [66]. While our dataset provides a valuable resource for use in future population-genetic studies, such as those requiring a non-human out-group [67, 68], a major underlying assumption is that human and chimpanzee genotypes at orthologous microsatellites are commensurable.

Microsatellite genotype data typically takes the form of polymerase chain reaction (PCR) fragment lengths obtained using locus-specific DNA primer pairs to amplify the specific genomic region encompassing a particular microsatellite in a collection of individuals. The rationale being that changes in PCR fragment length reflect changes in repeat numbers at STR regions embedded between the primer pair. Thus, differences in PCR fragment length are commonly used as a proxy for differences in repeat number. However, there are a number of caveats to this approach. Firstly, primer pairs are placed to optimize their PCR amplification efficiency rather than to satisfy specific distance criteria from the embedded STR regions. The distances of a primer pair from the embedded STR regions therefore vary markedly across microsatellites, and consequently PCR fragment lengths are incommensurable across microsatellites and do not allow absolute repeat numbers to be readily determined. Secondly, changes in PCR fragment length resulting from insertion/deletion (indel) events outside of the embedded STR regions cannot be distinguished from changes in repeat number in the STR regions [69–71]. This is particularly acute in interspecies comparisons that utilize a common set of primer pairs to genotype all species as non-STR sequences flanked by a primer pair may not be invariant across species [69–73].

Here, we identify the genomic targets of the primer pairs used to amplify the 246 human-derived autosomal microsatellites included in the combined human-chimpanzee dataset [66] in the chimpanzee reference sequence, and compare these chimpanzee sequences with their corresponding human sequences [74]. We investigate the extent of sequence differences at human and chimpanzee orthologs and their impact on human-chimpanzee comparisons based on two commonly used population-genetic statistics. Calibrating PCR fragment lengths against the human and chimpanzee reference sequences, we infer repeat number in individual genotypes and use the resulting dataset to perform the first direct comparison of microsatellite variability and its determinants at orthologous microsatellites in humans and chimpanzees with genotype data for many individuals.

## Results and discussion

### Identification and analysis of chimpanzee microsatellite sequences

**Figure 1:**
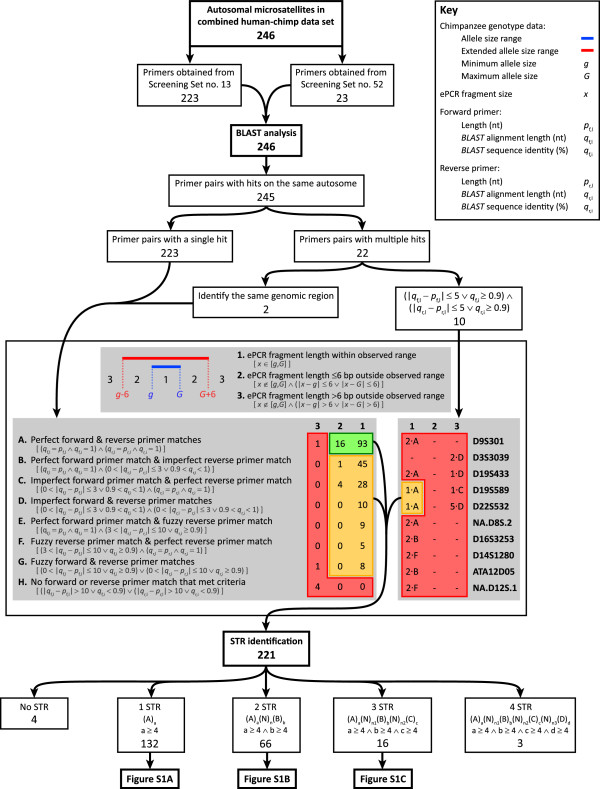
**Summary of the identification and sequence analysis of the chimpanzee microsatellite DNA sequences.** The blue bar indicates the PCR fragment length range in the chimpanzee genotype data, for which *g* and *G* are the smallest and largest PCR fragment lengths, respectively; the red bar represents the PCR fragment length range when extended by 6 bp on each side. The ePCR fragment length in the UCSC chimpanzee reference sequence is denoted by *x*. The quantities *p*_f,l_, *q*_f,l_ and *q*_f,i_ refer to the length, *BLASTN* alignment length, and *BLASTN* sequence identity of the forward primer, while *p*_r,l_, *q*_r,l_ and *q*_r,i_ refer to the exact same quantities for the reverse primer. *A*, *B*, *C*, *D* refer to the repeat units of the different STR regions in a microsatellite sequence, with *a*, *b*, *c*, and *d* being the number of times they are repeated, respectively. *N* indicates a nucleotide not within an STR region, with *n* being the number of nucleotides separating two consecutive STR regions. For microsatellites with three and four STR regions, *n*_1_, *n*_2,_ and *n*_3_ represent the numbers of nucleotides separating the first and second, the second and third, and the third and fourth STR regions, respectively. Key: ∧, and; ∨, or.

For all 246 autosomal microsatellites present in the human-chimpanzee dataset, putative PCR amplification targets were identified in release panTro4 of the UCSC chimpanzee reference genome sequence [75] using an *in silico* PCR (ePCR) approach [74] applied to DNA primer pairs obtained from the publicly available primer sequence files provided by the Mammalian Genotyping Service [76]. Despite the high level of sequence homology observed between the chimpanzee and human genomes [77], it was unlikely that the genomic targets of all human-derived primers would be perfectly conserved in the chimpanzee genome. Consequently, *BLASTN* “hits” for each primer were permitted to differ from its sequence by at most 10 nucleotides in alignment length and by at most 10% in sequence identity. Using these primer alignment criteria, putative autosomal target regions were identified for 245 of the 246 microsatellites (“ePCR fragments” henceforth). A single target region on chromosome Xq was identified by the primer pair of the remaining microsatellite (D1S3720); however, only 23 of the 48 male chimpanzees in the dataset had homozygous genotypes---contrary to what would be expected for an X-chromosomal marker---and it was consequently excluded from further analysis.To maximize the likelihood that the ePCR fragment identified for each microsatellite underlay the chimpanzee genotype data, its length was compared to the range of observed PCR fragment lengths. If an ePCR fragment’s length differed markedly from its corresponding range of PCR fragment lengths, there was a higher likelihood that the genomic region amplified by the primer pair had been incorrectly identified. Under the assumption that the genotype data captured the majority of length variability at each microsatellite, 14 microsatellites whose ePCR fragment length was more than 6 bp outside of their PCR fragment length range were excluded from further analysis (Figure 1). Six bp was a natural threshold: while there were a number of microsatellites with ePCR fragment lengths between 1 and 6 bp outside of the PCR fragment length range, all ePCR fragment lengths at these 14 microsatellites were at least 27 bp outside of this range. Of the 12 microsatellites whose DNA primer pair identified multiple ePCR fragments that met our primer alignment criteria, four were retained for further analysis. The primer pair for two of the four “multiple hit” microsatellites (D2S2972 and D4S2623) each identified two overlapping ePCR fragments that shared the same reverse primer position but had different forward primer positions. Both microsatellites were retained, with their target region defined as the smaller of the ePCR fragments under the assumption that it would be more efficiently amplified via PCR. Two further multiple hit microsatellites (D19S589 and D22S532) were retained because only one of their ePCR fragments met our length criteria; all of their other ePCR fragments had lengths at least 32 bp outside of their PCR fragment length range.

The repeat structure of the 221 microsatellites that met our criteria for retention was investigated, and STR regions---defined as runs of four or more contiguous repeats of a motif 2–6 nucleotides in length [78, 79]---were identified within their ePCR fragments (Additional file 1: Table S1). Four microsatellites (D10S1425, D13S779, D17S1294, and D20S164) had no STR regions identified within their ePCR fragment, while the remaining 217 had one (132), two (66), three (16), or four (3) STR regions comprised of di-, tri-, tetra-, or penta-nucleotide repeat units (Additional file 2: Figure S1).

### Comparison of fragment lengths at putative orthologs

**Figure 2:**
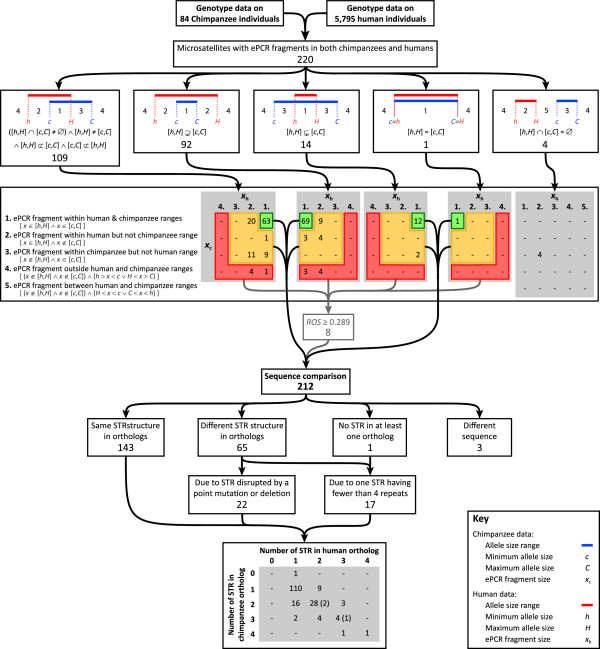
**Summary of interspecies comparisons of fragment lengths at putative orthologous microsatellites.** Blue bars indicate the PCR fragment length range in the chimpanzee genotype data, for which *c* and *C* are the smallest and largest PCR fragment lengths, respectively. Red bars indicate the PCR fragment length range in the human genotype data, for which *h* and *H* are the smallest and largest PCR fragment lengths, respectively. The ePCR fragment lengths in the chimpanzee and human reference sequences are denoted by *x*_c_ and *x*_h_, respectively. Key: ∧, and; ∨, or; *ROS*, range overlap score.

Under the null hypothesis of non-directional evolution, where in the absence of directional selection a microsatellite expands as often as it contracts within an infinite population, PCR fragment length distributions of orthologous microsatellites would be expected to be similar [80]. In this view, though microsatellites for which the chimpanzee and human PCR fragment length ranges do not overlap might reflect neutral genetic drift, they might also represent either PCR amplification of a non-orthologous region in the chimpanzee genome or different evolutionary constraints on the chimpanzee and human orthologs. In addition, under the assumption that the chimpanzee and human reference sequences are representative of a randomly sampled individual in the genotype dataset, ePCR fragment lengths would be expected to lie within the union of the chimpanzee and human PCR fragment length ranges. ePCR fragments that lie outside of their respective unified range had a lower likelihood of representing the genomic region amplified in the genotype data. Thus, at 220 of the 221 microsatellites whose chimpanzee ePCR fragments met our criteria for retention, the similarity of their chimpanzee and human PCR fragment length ranges in the genotype dataset was evaluated together with the location of their chimpanzee and previously reported human [74] ePCR fragment lengths within these ranges (Figure 2). One microsatellite (D1S1612) was excluded from further analysis as no human ePCR fragment was available.

Consistent with the null evolutionary hypothesis, chimpanzee and human PCR fragment length ranges overlapped for 216 of the 220 microsatellites. Four microsatellites (GATA51D11, D10S1425, D11S1999, and D13S779) whose chimpanzee and human ranges did not overlap were excluded from further analysis. Although their chimpanzee and human sequences were highly similar (data not shown), their chimpanzee range was shifted toward smaller lengths compared with their human range and their heterozygosities (*H*_e_) among chimpanzees (*H*_e_ < 0.155) was markedly lower than among humans (*H*_e_ > 0.626). Considered together, these observations suggested that these four microsatellites may be regressing in the chimpanzee genome. Compatible with this hypothesis, no STR regions were identified within the chimpanzee ePCR fragment of two of the four microsatellites (D10S1425 and D13S779).

At 92 of the 216 microsatellites with overlapping ranges, the chimpanzee range was subsumed by the human range, while the human range was subsumed by the chimpanzee range at only 14, likely reflecting the smaller number of chimpanzees (84) compared with humans (5795) in the dataset. Just a single microsatellite (D8S1108) had the exact same range in humans and in chimpanzees. The remaining 109 microsatellites had partially overlapping chimpanzee and human ranges, with the chimpanzee range shifted toward smaller lengths compared with the human range at 84, consistent with the observation that the majority of human-derived microsatellites are longer than their chimpanzee orthologs [81–85].

At the 216 microsatellites with overlapping chimpanzee and human ranges, chimpanzee and human ePCR fragment lengths were jointly investigated with respect to their unified chimpanzee-human range. Four microsatellites (D1S1609, D4S3243, D10S1225, and CATA002) whose chimpanzee ePCR fragment length fell outside of their unified range and range overlap scores (*ROS*) [74] of 0.286, 0.250, 0, and 0, respectively, were below 0.289---the lowest *ROS* among those microsatellites whose chimpanzee and human ePCR fragment lengths lay within the intersection of the chimpanzee and human ranges---were excluded from further analysis.

### Comparison of STR structure at putative orthologs

Microsatellite variability is known to be influenced by the number of distinct STR regions [47, 74, 86–89] as well as by their repeat unit size [25, 50, 74, 90–93] and motif [74, 94–96]. Thus, microsatellites whose chimpanzee and human amplicons contain discordant numbers of STR regions, or STR regions composed of different repeat unit sizes or motifs, might introduce interspecies incommensurability among genotypes. Therefore, at each of the 212 microsatellites whose ePCR fragment lengths and PCR fragment length ranges met our criteria for retention, the level of conservation of STR regions embedded within their chimpanzee and human ePCR fragments was evaluated.Of the 212 microsatellites compared, 143 had the same STR structure in both their chimpanzee and human ePCR fragments (Figure 2). Of the four microsatellites with no STR regions identified within their chimpanzee ePCR fragment, only one (D20S164) was included in the comparison and alignment of its chimpanzee and human ePCR fragments supported the presence of a single orthologous STR region comprised of only three repeats in the chimpanzee fragment and eleven repeats in the human fragment. Three microsatellites (D3S2427, D7S3056, and D17S1294) whose chimpanzee and human ePCR fragment sequences differed markedly (data not shown), potentially reflecting PCR amplification of a non-orthologous region in the chimpanzee genome or misidentification of the genomic region via ePCR, were excluded from further analysis.

Of the 65 microsatellites with different numbers of STR regions embedded in their chimpanzee and human ePCR fragments, 22 were the result of a point or indel mutation disrupting an otherwise orthologous STR region in either the chimpanzee (16) or human (6) ePCR fragment. Discordance at a further 36 microsatellites were the result of otherwise orthologous STR regions being comprised of only two or three repeats in either the chimpanzee (8 and 7, respectively) or human (8 and 9, respectively) ePCR fragment or both (4 and 0, respectively). Of these 65 microsatellites, 38 were retained for further analysis: the 16 and 22 microsatellites for which one ePCR fragment contained an otherwise orthologous STR region that was either comprised of only three repeats or disrupted by a mutation, respectively. The 20 microsatellites for which one ePCR fragment contained an otherwise orthologous STR region comprised of only two repeats, and seven microsatellites (D2S1360, D6S1277, D7S3070, TCTA017, D18S1357, D19S589, and NA.D22S.1) that had one or more discordant STR regions in their chimpanzee and human ePCR fragments, were excluded from further analysis. Intriguingly, at three of these seven microsatellites, a different motif had expanded in chimpanzees and humans to form an otherwise identically positioned STR region. At two of these microsatellites, the motifs differed by just a single position---CTGT/CTGC (D7S3070) and CTA/ATA (D18S1357)---suggesting that an ancient point mutation in either the chimpanzee or human lineage might underlie this difference; at the remaining microsatellite (D2S1360) the motifs differed more markedly (TACC/TGTC).

### Comparison of ortholog heterozygosities

**Figure 3:**
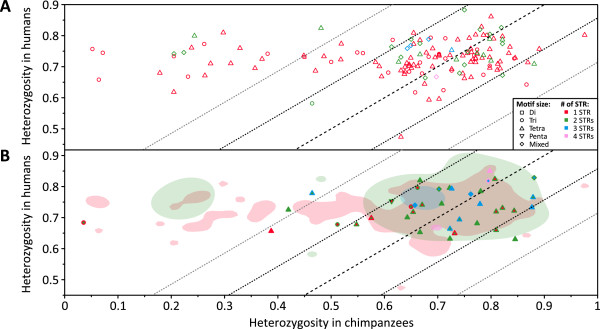
**Interspecies comparison of heterozygosities at orthologous microsatellites.** Scatterplots comparing *H*_e_ at orthologous microsatellites in chimpanzees and humans are shown for **(A)** 143 microsatellites where the human and chimpanzee ePCR fragments had the same STR structure, and **(B)** 39 microsatellites where one or more STR regions in the human or chimpanzee ePCR fragment were comprised of only three repeats or disrupted by a mutation overlaid on the 92% utilization distributions for the microsatellites in **A**. Each symbol’s line and fill color indicate the number of STR regions in the chimpanzee and human ePCR fragments, respectively, in **B**. Black dashed lines depict the identity line, and black and grey dotted lines depict ±1 SD and ±2 SD departures from the identity line, respectively.

Investigation of *H*_e_ in the human-chimpanzee dataset for the 182 microsatellites that had either the same STR structure in both the chimpanzee and human ePCR fragments (143) or had an otherwise orthologous STR region comprised of only three repeats (17) or disrupted by a mutation (22) in one ePCR fragment found that the majority of orthologs generally had similar *H*_e_ in chimpanzees and humans (Figure 3). Among the 143 microsatellites for which the human and chimpanzee ePCR fragments had the same STR structure (Figure 3A), 104 had *H*_e_ in chimpanzees and humans that were significantly positively correlated (Spearman *ρ* = 0.269, *P* = 0.005) and lay within 1 SD---calculated across all 182 microsatellites---of the identity line. The positive correlation remained significant when restricted to the 58 microsatellites with one tetranucleotide STR region (*ρ* = 0.372, *P* = 0.004), but not when restricted to the 19 microsatellites with one trinucleotide STR region (*ρ* = 0.214, *P* = 0.363) or to the 8 tetranucleotide and 14 mixed-repeat-unit-size microsatellites with two STR regions (*ρ* = 0.476 with *P* = 0.363 and *ρ* = 0.174 with *P* = 0.548, respectively) despite their *H*_e_ in chimpanzees and humans not being significantly different (average relative difference [ARD] = 1.001 with standard deviation [SD] = 0.104 and *P* = 0.985, ARD = 1.008 with SD = 0.081 and *P* = 0.641, and ARD = 0.971 with SD = 0.120 and *P* = 0.502, respectively; Wilcoxon signed-rank test). The generally similar *H*_e_ in chimpanzees and humans is at odds with effective population size (*N*_*e*_) estimates, where the larger *N*_*e*_ of chimpanzees [97, 98] compared with humans [99, 100] would be expected to confer higher *H*_e_ [101]. However, it is compatible with a scenario in which the effects of *N*_*e*_ are largely abrogated by reduced mutability at human-derived microsatellites in chimpanzees compared with humans due to their generally shorter lengths [41, 81–85, 96, 102–104].

Interestingly, 21 microsatellites had markedly lower *H*_e_---more than 2 SD below the identity line---in chimpanzees than in humans (ARD = 0.328 with SD = 0.130, *P* = 1.91 × 10^-6^; “outlier microsatellites” henceforth). Focusing on the 7 tri- and 10 tetra-nucleotide single STR outlier microsatellites, their chimpanzee *H*_e_ were significantly lower than those of the 24 tri- and 68 tetra-nucleotide single STR non-outlier microsatellites, respectively, while their human *H*_e_ were not significantly different (Table 1). Comparison of the distributions of repeat numbers at outlier and non-outlier microsatellites found that the outliers had significantly lower mean, maximum, and minimum numbers of repeats than the non-outliers in chimpanzees but not in humans (Table 1). Further, the distributions of repeat numbers at the outlier microsatellites were significantly more positively skewed than those at the non-outlier microsatellites in chimpanzees but not in humans (Table 1). These findings accord with those of prior studies that identified positive correlations between mean and maximum numbers of repeats and *H*_e_ [74, 94] as well as observed mutations at microsatellites with smaller numbers of repeats to be biased toward expansion [34, 105] and to increase in frequency as a function of repeat number [64, 65] owing to peculiarities in DNA replication mismatch repair processes.

**Table 1:** **Comparison of heterozygosity and measures of variation across individuals at outlier and non**-**outlier microsatellites**

Variable	Chimpanzee				Human			
	Tri		Tetra		Tri		Tetra	
	RDM	*P*	RDM	*P*	RDM	*P*	RDM	*P*
Heterozygosity (*H*_e_)	0.258	**7.97 × 10** ^**-5**^	0.395	**3.88 × 10** ^**-7**^	1.010	0.595	0.986	0.469
Number of distinct alleles	0.532	**2.47 × 10** ^**-4**^	0.795	0.150	0.999	0.444	1.046	0.299
Variance in the number of repeats	0.293	**0.001**	0.868	0.122	1.298	0.104	1.314	0.958
Range of the number of repeats	0.556	**0.001**	0.865	0.722	1.050	0.274	1.050	0.279
Skewness in the number of repeats	4.549	**0.012**	3.349	**0.004**	0.650	0.627	1.331	0.606
Mean PCR fragment length	0.898	0.365	0.856	0.167	0.932	0.532	0.953	0.737
Mean number of repeats	0.637	**2.28 × 10** ^**-5**^	0.558	**1.06 × 10** ^**-6**^	0.882	0.094	0.937	0.223
Maximum number of repeats	0.654	**1.78 × 10** ^**-4**^	0.755	**7.50 × 10** ^**-4**^	0.940	0.274	0.953	0.596
Minimum number of repeats	0.823	**0.038**	0.628	**7.35 × 10** ^**-4**^	0.838	**0.029**	0.859	0.087

Wilcoxon rank sum tests are shown for comparisons of continuous microsatellite sequence properties between the 7 tri- and 10 tetra-nucleotide single STR outlier microsatellites and the 24 and 68 single STR tri- and tetra-nucleotide non-outlier microsatellites. Relative difference in means (RDM) was calculated by dividing the mean of the variable among the outlier microsatellites by the mean of the variable among the non-outlier microsatellites. *P* < 0.05 are shown in **bold**.

At the 39 microsatellites for which the human and chimpanzee ePCR fragments had discordant STR structures owing to an STR region being either comprised of just three repeats (17) or disrupted by a mutation (22) in one ePCR fragment, *H*_e_ were found to be highly similar to those of the 143 microsatellites whose human and chimpanzee ePCR fragments had the same STR structure (Figure 3B). *H*_e_ for the 17 microsatellites with an STR region comprised of only three repeats in either their chimpanzee (8) or human (9) ePCR fragment did not differ significantly between species (ARD = 0.962 with SD = 0.193; *P* = 0.378, Wilcoxon signed-rank test). Considering just the 10 microsatellites with two tetranucleotide STR regions among these 17---the only group with a sample size greater than three---their *H*_e_ did not differ significantly from the 10 microsatellites with two tetranucleotide STR regions comprised of four or more repeats in chimpanzees (relative difference in means [RDM] = 0.943; *P* = 0.256, Wilcoxon rank sum test) or in humans (RDM = 0.957, *P* = 0.315). These findings support the retention of these 17 microsatellites for future analyses. While potentially discordant with prior observations that indicated four or more repeats were required for microsatellite mutability [78, 79], the three repeats present in the reference sequence might reflect truncations during reference sequence assembly or an unusually low number of repeats in the individual(s) used to generate this sequence.

*H*_e_ for the 22 microsatellites with an otherwise orthologous STR region disrupted by a mutation in either the chimpanzee (16) or human (6) ePCR fragment did not differ significantly between species (ARD = 0.925 with SD = 0.253; *P* = 0.235, Wilcoxon signed-rank test). Considering the nine tetranucleotide microsatellites with two STR regions in chimpanzees but one STR region in humans, their *H*_e_ in chimpanzees were not significantly different from those of the 68 and 10 tetranucleotide microsatellites with one (RDM = 1.042; *P* = 0.236, Wilcoxon rank sum test) or two (RDM = 1.012, *P* = 0.534) STR regions, respectively, that had the same STR structure in both species. Additionally, the four tetranucleotide microsatellites with three STRs in chimpanzees but two STR regions in humans had *H*_e_ in chimpanzees that were not appreciably different from the 10 microsatellites with two tetranucleotide STR regions (RDM = 0.952, *P* = 0.635). Our findings with interrupted and uninterrupted arrays of tetranucleotide repeats therefore disagree with those of prior studies that found uninterrupted arrays of di- and tri-nucleotide repeats to be more polymorphic than those with interruptions both in genomic DNA [47, 86, 88] and plasmid construct [87, 89] environments. This discordance might reflect different tolerances to disruption by mutations of STR regions comprised of tetranucleotide repeats compared with di- and tri-nucleotide repeats. However, we could not discount the possibility that some of the observed mutations might be rare variants captured in the reference sequence but largely absent in the general population.

### Impact of fragment length imbalances on estimates of genetic differentiation

During the alignment of chimpanzee and human sequences, it became apparent that nontrivial ePCR fragment length differences that are not the result of embedded STR regions existed, in accord with earlier studies of much smaller sets of microsatellites [71, 72]. To investigate this further, at the 182 microsatellites that had either the same STR structure in both the chimpanzee and human ePCR fragments when considering STR regions with three or more repeats (160) or had an otherwise orthologous STR region disrupted by a mutation in one ePCR fragment (22), the chimpanzee and human ePCR fragment sequences were manually aligned and the number of nucleotides present in only one ePCR fragment was tabulated (Additional file 3: Table S2).

**Figure 4:**
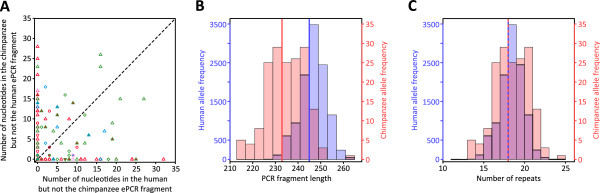
**Interspecies comparison of non**-**STR ePCR fragment length differences. (A)** A scatterplot comparing the number of nucleotides outside of STR regions that are present in either the human or chimpanzee ePCR fragment but not both. Symbols and colors follow Figure 3. For microsatellite NA.D12S.2 the distribution of alleles in humans (blue) and in chimpanzees (red) are shown for **(B)** PCR fragment lengths and **(C)** repeat numbers. The blue and red vertical lines indicate the mean length in humans and chimpanzees, respectively.

Non-STR length differences at some microsatellites were appreciable (Figure 4A), contributing up to an additional 28 nucleotides to the chimpanzee ePCR fragment and 32 nucleotides to the human ePCR fragment. The magnitude of non-STR length differences was observed to decrease as a function of average STR length across individuals for microsatellites with one or two STR regions (*r* = -0.163 with *P* = 0.044 and *r* = -0.586 with *P* = 1.81 × 10^-5^, respectively), consistent with the inverse relationship reported between flanking sequence divergence and STR length at orthologous mammalian microsatellites [106]. Such large differences create incommensurability between chimpanzee and human PCR fragment lengths, as chimpanzee and human PCR fragments of the same length may reflect different numbers of repeats. For example, the distributions of human and chimpanzee PCR fragment lengths at microsatellite NA.D12S.2 were discordant (Figure 4B), with the chimpanzee distribution shifted toward smaller lengths than the human distribution. However, when the distributions of the repeat numbers those PCR fragment lengths represent were compared the human and chimpanzee distributions were instead highly similar (Figure 4C). Conversely, at microsatellite D6S2410, while the distributions of human and chimpanzee PCR fragment lengths were similar (Additional file 4: Figure S2A), the distributions of repeat numbers were instead largely discrete (Additional file 4: Figure S2B). Thus, the use of PCR fragment lengths in lieu of the repeat numbers they represent at orthologous chimpanzee and human microsatellites had the potential to detrimentally bias interspecies comparisons.

To evaluate the extent to which chimpanzee and human PCR fragment length incommensurability might impact interspecies comparisons, pairwise estimates of the fixation index (*F*_ST_) and allele-sharing distance (*D*_PS_)---one minus the proportion of shared alleles---calculated on the basis of PCR fragment lengths and repeat numbers were compared. For these analyses, only the 138 microsatellites whose chimpanzee and human ePCR fragments had the same STR structure comprised of repeat units of a single size were retained. The 22 microsatellites for which an otherwise orthologous STR region was disrupted by a mutation in one ePCR fragment, as well as the 22 microsatellites containing two, three, or four STR regions comprised of repeat units of different sizes, were excluded because of the resulting difficulty in assigning repeat number. Under the assumption that within-species variability in PCR fragment lengths in the human-chimpanzee dataset are wholly due to changes in repeat number in embedded STR regions, PCR fragment length genotypes were converted into the repeat numbers they represent via calibration against their chimpanzee and human ePCR fragments (Additional file 5).

**Figure 5:**
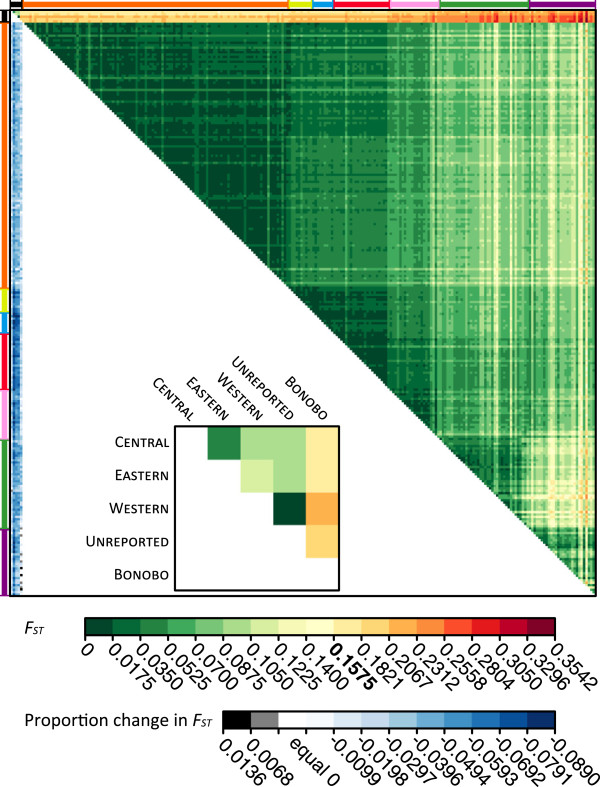
**Comparison of pairwise** ***F***_**ST**_ **calculated on the basis of PCR fragment lengths and repeat numbers.** A heatmap of pairwise *F*_ST_ values among the 243 non-admixed human and five chimpanzee populations with a sample size of at least five individuals in the human-chimpanzee dataset (upper triangle) and the proportion change in *F*_ST_ when values are calculated using repeat numbers instead of PCR fragment lengths (lower triangle). Chimpanzee populations are located on the top-left of the plot (black bars), with the human populations ordered from top to bottom and from left to right by geographic affiliation, as indicated by colored bars (orange, Africa; yellow, the Middle East; blue, Europe; red, Central/South Asia; pink, East Asia; green, Oceania; purple, the Americas), and within regions from top to bottom and from left to right by increasing geographic distance from Addis Ababa.

Next, separately for PCR fragment lengths and repeat numbers, pairwise *F*_ST_ was calculated among the 243 non-admixed human and five chimpanzee populations in the dataset with a sample size of at least five individuals (Figure 5). While *F*_ST_ values calculated on the basis of PCR fragment lengths and repeat numbers were highly correlated (*R*^*2*^ = 0.999), interspecies *F*_ST_ values calculated on the basis of repeat numbers were significantly lower than those calculated on the basis of PCR fragment lengths (*P* < 10^-16^, Wilcoxon signed rank test). Across all interspecies comparisons, a maximal *F*_ST_ reduction of 8.90% was observed (mean = 4.03%, SD = 1.74%) with the magnitude of the reduction generally increasing with the distance of the human population from Addis Ababa, Ethiopia, a reasonable proxy for the origin of the out-of-Africa migration of AMH (*ρ* = 0.454, *P* = 2.94 × 10^-8^). If *F*_ST_ values calculated on the basis of repeat numbers at these 138 microsatellites were instead compared with those calculated on the basis of PCR fragment lengths at all 246 microsatellites in the human-chimpanzee dataset analogous reductions were observed (max = 10.6%, mean = 4.82%, SD = 2.13%). Similar patterns were observed when pairwise *D*_PS_ values were compared (Additional file 6: Figure S3).

These results are compatible with a scenario in which non-STR length differences lead to the misalignment of orthologous human and chimpanzee alleles when they are represented by PCR fragment lengths, distorting numbers of shared and private alleles in interspecies comparisons. This distortion is magnified by natural increases in numbers of private alleles as a function of the human population’s distance from Africa [107, 108], owing to the concomitant reduction in genetic diversity [20, 22, 66, 109] as well as increases in population isolation [110] and human-chimpanzee dissimilarity (Figure 5 and Additional file 6: Figure S3). In this view, the cumulative effects of distortions across microsatellites inflated levels of human-chimpanzee genetic differentiation compared with those obtained with repeat numbers, with comparisons for human populations more distant from Africa affected to a greater extent owing to their naturally higher numbers of private alleles.

### Interspecies differences in microsatellite properties and their effects on heterozygosity

Our human-chimpanzee dataset comprised of 138 microsatellites with the same STR structure in humans and chimpanzees and genotypes represented as repeat numbers (Additional file 5) afforded us the first opportunity to directly compare and contrast determinants of variability at orthologous microsatellites in humans and chimpanzees with genotype data on many individuals. In these comparisons, the 138 microsatellites were grouped by the number of distinct STR regions embedded in their sequence and by their repeat unit size. For each of the 138 microsatellites, the values of investigated measures can be found in Table S3 (Additional file 7), and a summary of the mean, minimum, and maximum values across microsatellites in each group appears in Table S4 (Additional file 8).

#### Interspecies differences in microsatellite properties

In agreement with an earlier study of 19 orthologous dinucleotide microsatellites in chimpanzees and humans [82], chimpanzee microsatellites were found to generally have significantly larger numbers of distinct alleles than their human orthologs (Table 2). Consistent with this observation, chimpanzee microsatellites were also observed to generally have significantly larger ranges of repeat numbers than their human orthologs (Table 2). These findings accord with the substantially higher levels of genetic diversity observed among chimpanzees than among humans [111–113], reflecting the larger effective population size of chimpanzees [97, 98] compared with humans [99, 100]. Despite the higher numbers of distinct alleles present in chimpanzees, variance in repeat number was generally similar in chimpanzees and humans (Table 2). This would suggest that the majority of chimpanzee-specific alleles lie in the tails of the repeat number distribution, in agreement with observed levels of human-chimpanzee differentiation (Figure 5 and Additional file 6: Figure S3) as well as the reported relationship between inter-population levels of genetic differentiation and the location of private alleles within their allele size distributions [108].

**Table 2:** **Comparison of measures of variation across individuals at orthologous microsatellites**

Variable	1 STR region				2 STR regions	
	Tri		Tetra		Tetra	
	*n* = 31		*n* = 79		*n* = 21	
	ARD (SD)	*P*	ARD (SD)	*P*	ARD (SD)	*P*
Number of distinct alleles	0.949 (0.366)	0.264	1.257 (0.448)	**3.73** ** × ** **10** ^**-6**^	1.508 (0.477)	**1.97** ** × ** **10** ^**-4**^
Variance in the number of repeats	1.025 (1.030)	0.120	1.813 (1.623)	**8.08** ** × ** **10** ^**-4**^	2.413 (2.401)	0.065
Range of the number of repeats	0.915 (0.336)	0.157	1.167 (0.336)	**5.65** ** × ** **10** ^**-5**^	1.302 (0.354)	**0.011**
Mean PCR fragment length	0.962 (0.045)	**3.18** ** × ** **10** ^**-5**^	0.965 (0.056)	**7.12** ** × ** **10** ^**-7**^	0.951 (0.064)	**0.003**
Mean number of repeats	0.793 (0.220)	**1.05** ** × ** **10** ^**-5**^	0.875 (0.230)	**4.07** ** × ** **10** ^**-7**^	0.861 (0.189)	**0.001**
Maximum number of repeats	0.830 (0.178)	**2.99** ** × ** **10** ^**-6**^	0.967 (0.197)	**0.022**	0.959 (0.170)	0.495
Minimum number of repeats	0.794 (0.210)	**5.08** ** × ** **10** ^**-5**^	0.887 (0.639)	**6.44** ** × ** **10** ^**-7**^	0.734 (0.263)	**5.25** ** × ** **10** ^**-5**^

Wilcoxon signed-rank tests are shown for comparisons of measures of variation across individuals calculated separately among chimpanzees and humans. Microsatellites were grouped by their number of separate STR regions and repeat unit size. No comparisons were performed for microsatellites with one dinucleotide STR region, two trinucleotide STR regions, or three or four tetranucleotide STR regions because of small sample sizes (1, 1, 4, and 1, respectively). Average relative difference (ARD) was calculated by taking the average across microsatellites of the division of the value in chimpanzees by the value in humans. The standard deviation (SD) in relative difference across microsatellites is provided in parentheses. *P* < 0.05 are shown in **bold**.

Mean PCR fragment lengths in chimpanzees were significantly shorter than those in humans (Table 2). These findings were not wholly a consequence of non-STR length differences between chimpanzee and human PCR amplicons (*P* > 0.212, Wilcoxon signed-rank test), suggesting that chimpanzee microsatellites contain fewer repeats than their human orthologs on average. Indeed, comparison of repeat number distributions at orthologous microsatellites found that chimpanzees generally had significantly lower mean, maximum, and minimum numbers of repeats than humans (Table 2). Across the 138 microsatellites in our dataset, the human ortholog was on average 2.04 repeats longer than the chimpanzee ortholog, similar to the value of 1.97 reported for 47 dinucleotide microsatellites genotyped in six chimpanzees and six humans [82], but slightly smaller than the value of 2.31 determined via comparative genomics [84]. Thus, our findings further support the view that human-derived microsatellites generally have greater repeat numbers than their chimpanzee orthologs [81–84], potentially reflecting interspecies variability in directional biases in the mutation process [84], the biological and evolutionary bases of which remain enigmatic.

#### Effect of microsatellite properties on heterozygosity

In agreement with our earlier study [74], the number of distinct alleles, the range of repeat numbers, and the variance in repeat number were generally positively correlated with *H*_e_ in both chimpanzees and humans (Table 3). Similarly, in accord with prior studies in humans [32, 74] and *Drosophila melanogaster* [86, 94], the mean and maximum numbers of repeats were also generally positively correlated with *H*_e_ in both chimpanzees and humans (Table 3). However, consistent with previous studies [74, 114], the minimum number of repeats and mean PCR fragment length were generally not significantly correlated with *H*_e_ in either chimpanzees or humans (Table 3). While some of these observations might arise from a general correlation among the various measures (Additional file 9: Table S5), how can their patterns be explained in terms of their relationship to the microsatellite replication slippage mutation mechanism [41–45]?

**Table 3:** **Relationship of microsatellite heterozygosity with measures of variation across individuals**

Variable		1 STR region				2 STR regions	
		Tri		Tetra		Tetra	
		*n* = 31		*n* = 79		*n* = 21	
		Chimpanzee	Human	Chimpanzee	Human	Chimpanzee	Human
Number of distinct alleles	*ρ*	**0.643**	0.207	**0.292**	**0.657**	0.301	**0.694**
	*P*	**9.51 × ** **10** ^**-5**^	0.263	**0.009**	**4.97 × ** **10** ^**-11**^	0.184	**6.89 × ** **10** ^**-4**^
Variance in the number of repeats	*ρ*	**0.594**	0.240	**0.336**	**0.730**	**0.596**	**0.851**
	*P*	**4.26 × ** **10** ^**-4**^	0.194	**0.002**	<**10** ^-**16**^	**0.004**	<**10** ^-**16**^
Range of the number of repeats	*ρ*	**0.433**	0.152	0.166	**0.602**	**0.440**	**0.784**
	*P*	**0.015**	0.411	0.144	**4.35 × ** **10** ^**-9**^	**0.046**	**3.62 × ** **10** ^**-5**^
Mean PCR fragment length	*ρ*	0.093	0.044	**0.230**	0.084	0.096	-0.134
	*P*	0.617	0.814	**0.042**	0.461	0.676	0.562
Mean number of repeats	*ρ*	**0.529**	**0.388**	**0.461**	0.184	**0.522**	**0.526**
	*P*	**0.002**	**0.032**	**1.88 × ** **10** ^**-5**^	0.105	**0.015**	**0.016**
Maximum number of repeats	*ρ*	**0.405**	0.216	**0.391**	**0.386**	**0.632**	**0.635**
	*P*	**0.024**	0.243	**3.68** ** × 10** ^**-4**^	**4.82** ** × 10** ^**-4**^	**0.002**	**0.002**
Minimum number of repeats	*ρ*	0.068	0.136	**0.326**	0.046	0.122	-0.156
	*P*	0.717	0.464	**0.003**	0.686	0.598	0.498

Spearman’s rank correlation coefficients (*ρ*) are shown for comparisons of microsatellite *H*_e_ with measures of variation across individuals calculated separately among chimpanzees and humans. Microsatellites were grouped by their number of separate STR regions and repeat unit size. No comparisons were performed for microsatellites with one dinucleotide STR region, two trinucleotide STR regions, or three or four tetranucleotide STR regions because of small sample sizes (1, 1, 4, and 1, respectively). Correlations with *P* < 0.05 are shown in **bold**.

Replication slippage occurs because of homology among microsatellite repeats, providing the opportunity for the two DNA strands to realign incorrectly after polymerase dissociation and strand separation, introducing a loop in one strand and leading to microsatellite expansion or contraction after the resumption of replication [43, 45, 115]. The direct relationships observed between the mean and maximum numbers of repeats and *H*_e_ are consistent with a scenario in which the probability of slipped-strand mispairing during DNA replication increases as a function of repeat number, with a concomitant increase in the probability of microsatellite mutation [35, 41, 93, 104, 116, 117]. The absence of a similar relationship between *H*_e_ and the minimum number of repeats is compatible with the idea that the minimum number of repeats, while an important predictor of the lower bound of microsatellite mutability [78, 79], is not informative about overall levels of mutability. The direct relationships observed between *H*_e_ and the number of distinct alleles, the range of repeat numbers, and the variance in repeat number accord with the increased probability of observing heterozygous genotypes as a function of the number of available alleles and the concomitant increases in their range and variance. Finally, as a consequence of PCR primer pairs being positioned to optimize their amplification efficiency rather than to satisfy specific distance criteria from the embedded STR regions, the absence of a relationship between mean PCR fragment length and *H*_e_ accords with the view that PCR fragment lengths are not comparable in a meaningful way across microsatellites.

## Conclusions

By identifying and comparing the genomic sequence and repeat structure of orthologous human and chimpanzee PCR amplicons that underlie the genotypes in the largest human-chimpanzee microsatellite dataset of its kind to date, our study provides new insights into the parallel evolution of orthologous microsatellites used in population-genetic studies for over a decade. Our results demonstrate that human-chimpanzee differences within the flanking sequences of embedded STR regions are frequent and can introduce non-trivial length imbalances into their PCR amplicons. The latter observation is important as it creates incommensurability among chimpanzee and human PCR fragment length genotypes, which we show can inflate the commonly used *F*_ST_ and *D*_PS_ population-genetic statistics by up to ~10.6%. Our results therefore suggest that the findings of prior interspecies comparisons based upon PCR fragment length genotypes derived from a common set of DNA primer pairs (e.g. [25, 81, 82, 118–120]) should be interpreted with caution, as the extent to which non-STR length differences contributed to their observed patterns remains unknown. Furthermore, they indicate that future interspecies studies utilizing microsatellites should implement the approach we describe here, based either on available reference genome sequences or microsatellite-specific sequencing in a small number of individuals, to avoid potential pitfalls stemming from PCR fragment length incommensurability among species.

To overcome sequence-derived incommensurability among human and chimpanzee genotypes in the human-chimpanzee dataset, we use the human and chimpanzee reference sequences for 138 microsatellites whose STR structure we found to be identical in both species to convert their PCR fragment length genotypes into the repeat numbers they represent (Additional file 5). This resource offers new opportunities for more accurate comparisons of patterns of human and chimpanzee genetic variation in numerous areas of application than were possible with earlier datasets. While future studies jointly investigating millions of orthologous human and chimpanzee SNPs should have greater power to resolve fine-scale interspecies relationships than our dataset of 138 microsatellites, frequent homoplasy at orthologous positions in the human and chimpanzee genomes [121] poses a significant challenge in developing the necessary resources for such undertakings. Moreover, the findings of recent studies would suggest the joint investigation of future SNP datasets together with our dataset of 138 “gold standard” microsatellites may afford future studies a more complete view of intergroup relationships than can be obtained from analyses of either marker type alone [54, 122, 123].

Finally, our study provides the first direct comparison of determinants of variability at orthologous microsatellites in humans and chimpanzees, jointly considering sequence properties together with measures of genetic diversity among human and chimpanzee populations. Although it is important to note that we have not sequenced these human-derived microsatellites in each individual, and have instead assumed that PCR fragment length differences are wholly due to changes in their embedded STR regions, we have no reason to suspect that these issues might have systematically affected the particular comparisons we have performed.

## Methods

### Genotype data

The analyzed dataset consisted of the MS5879 subset of the Pemberton *et al*. [66] human-chimpanzee dataset that contains genotypes at 246 autosomal microsatellites in 5,795 individuals from 267 human populations and 84 individuals from six chimpanzee groups. These genotype data consist of PCR fragment lengths at each microsatellite in each individual. Geographic region assignments and geographic distances from Addis Ababa for the human populations follow Pemberton *et al*. [66].

### DNA primer pairs

The 246 microsatellites were comprised of 223 from Marshfield Screening Set no. 13 and 23 from Marshfield Screening Sets no. 52 [65]. Primer pairs for all 246 microsatellites were obtained from the publicly available primer sequence files provided by the Mammalian Genotyping Service [76] (Marshfield Clinic, Marshfield, WI) for the Screening Set from which their genotypes were obtained with one exception. Both the forward and reverse primers provided for microsatellite D2S1394 (GATA69E12) in Screening Set no. 13 were identical except for the addition of a single A nucleotide to the 5’ end of the reverse primer, and their sequence matched that of the reverse primer reported in Pemberton *et al*. [74] obtained from Screening Set no. 10. As this discrepancy likely reflects an error in the primer sequence file provided for Screening Set no. 13, the primer pair for D2S1394 was instead taken from Screening Set no. 10. The primer pairs used in this study can be found in Table S1 (Additional file 1).

### Identification of chimpanzee genomic targets

The ePCR analysis pipeline was adapted from Pemberton *et al*. [74]. First, the sequence of each forward primer and each reverse primer was separately used as the query in *BLASTN* searches of release panTro4 of the UCSC chimpanzee reference sequence using the standalone *blastall* application (v.2.2.26) [124] with the repetitive sequence filter turned off and the expected value set to 1000. For those primers listed in the Marshfield Screening Set as having been modified with a 7 bp pig-tail [125] or with a single extra adenine base---identified by a one letter suffix, P or M, respectively, in the Marshfield marker name---we included the non-genomic sequence in the *BLASTN* search but not in the assessment of alignment length.

Next, to identify the most probable PCR amplicon for each microsatellite, the length of the ePCR fragments demarcated by all possible pairs of forward and reverse primer “hits” from the same chromosome was calculated as the distance between the terminal 5′ nucleotide of the forward primer “hit” and the terminal 5′ nucleotide of the reverse primer “hit.” These ePCR fragment lengths were then compared against the corresponding PCR fragment length range among the 84 chimpanzees in the genotype dataset. If the forward or reverse primer used to genotype a microsatellite had been modified with a 7 bp pig-tail or with a single extra adenine base, the size of the ePCR fragment demarcated by the primer pair was adjusted by the addition of 7 bp or 1 bp, respectively, prior to comparison with the range. In addition, when the chimpanzee and human datasets were merged, all chimpanzee genotypes at select microsatellites were adjusted by the same amount to account for primer differences among the constituent datasets [66]. At such microsatellites, these adjustments were reversed prior to comparison.

For a microsatellite to be flagged as “found,” the length of the ePCR fragment demarcated by the forward primer “hit” and the reverse primer “hit” was required to lie either: (i) within the PCR fragment length range among the 84 chimpanzees in the genotype dataset, or (ii) at most 6 bp outside the PCR fragment length range to account for the possibility that the samples used to define the range might not capture the full range of chimpanzee diversity, causing the ePCR fragment length to fall just outside the range. If the length of the demarcated ePCR fragment met one of the two criteria, its sequence was extracted from the reference sequence using the *fastacmd* application (v.2.2.26).

### Analysis of chimpanzee microsatellite sequences

Based upon the observation that for STR regions with a repeat unit of 2–6 nucleotides four or more contiguous repeats are required for polymorphism [78, 79], only STR regions that met this criteria were considered; a microsatellite can contain one or more STR regions embedded between the primer pair used to amplify it. All contiguous repeats of the same motif were considered part of an STR region, and a single interruption of one base pair or greater in a run of contiguous repeats as a break in the repeat structure with contiguous runs on either side of the interruption treated as separate STR regions provided each was comprised of at least four repeats. For each microsatellite, all STR regions were identified in its ePCR fragment sequence and the total number of repeats was tabulated. If more than one STR region was detected, whether or not the STR regions shared a common repeat motif was determined. As multiple STR regions may have arisen through interruptions in a single ancestral STR region, the boundaries of the STR regions were shifted such that they shared a common repeat motif, where possible, provided the number of repeats remained the same. Mosaic plot [126, 127] representations of contingency tables of microsatellite categories were created using *mosaic* from the *vcd* package [128] in the R statistical software program (v.3.0.0) [129].

### Comparison of human and chimpanzee microsatellite sequences

Human ePCR fragments were obtained from Pemberton *et al*. [74]. For each microsatellite, PCR fragment length ranges among the 84 chimpanzees and among the 5,795 humans in the genotype dataset were jointly compared with their corresponding ePCR fragment lengths, correcting for the microsatellite-specific PCR fragment length adjustments performed by Pemberton *et al*. [66]. The chimpanzee and human ePCR fragments were considered orthologs if they met one of three criteria: (i) Both chimpanzee and human ePCR fragment lengths lay within the intersection of the chimpanzee and human PCR fragment length ranges computed from the genotype dataset; (ii) Either the chimpanzee or human ePCR fragment length lay outside of their corresponding range but was within the union of the chimpanzee and human ranges; (iii) If the chimpanzee or human ePCR fragment length lay outside of the union of the chimpanzee and human ranges, then the *ROS* of the chimpanzee and human ranges was calculated as previously described [74]. A situation that met criterion (iii) might arise if the samples used to define the chimpanzee and human ranges do not fully capture the range of diversity at the microsatellite. If a microsatellite had *ROS* ≥0.289, then the chimpanzee and human ePCR fragments were considered orthologs provided the offending ePCR fragment was not more than 6 bp outside of the unified range.

To evaluate levels of conservation among embedded STR regions and their flanking sequences, the chimpanzee and human ePCR fragment sequences were manually aligned for each microsatellite, allowing for occasional point mutations and small indels. Only repeat units with identical motifs were permitted to align within an STR region; the rest of the sequence was considered non-polymorphic flanking sequence under the assumption that it was unlikely to participate in the microsatellite mutation process. For simplicity, all indels were treated as insertions in the respective ePCR fragment.

### Conversion of PCR fragment lengths into repeat numbers

Under the assumption that differences in PCR fragment length are exclusively the result of differences in repeat number at embedded STR regions, PCR fragment lengths in individual human and chimpanzee genotypes were calibrated against their respective reference sequence to infer repeat number in the genotype dataset as previously described [74]. At some microsatellites, all repeat number genotypes were non-integer and had a common decimal fraction (e.g. for a tetranucleotide repeat unit all genotypes had a decimal fraction of 0.75), potentially reflecting small inaccuracies in the reference sequences or genotype calls. In these cases, all genotypes were rounded to the nearest integer value. Repeat number genotypes at microsatellites where multiple decimal fractions were observed were not adjusted.

### Calculation of population-genetic statistics

Separately for each microsatellite, levels of variability among the 84 chimpanzee individuals and among the 5,435 human individuals in the MS5519 subset [66] of the genotype dataset were evaluated with *H*_e_ calculated using a sample size-corrected estimator [130], considering in the calculation only those individuals with non-missing genotypes. Levels of differentiation among the 243 non-admixed human and five chimpanzee populations with a sample size of at least five individuals in the MS5519 subset were evaluated using the *F*_ST_ and *D*_PS_ estimators. Separately for genotypes represented as PCR fragment lengths and as repeat numbers, *F*_*ST*_ and *D*_*PS*_ estimates were computed between all possible population pairs using *Arlequin* (v.3.5.1.3) [131] and *microsat* [132], respectively.

### Analysis of microsatellite diversity data

Statistical analyses were performed in R. In the scatterplot comparing *H*_e_ in humans and chimpanzees, *kernelUD* from the *adehabitatHR* package [133] was used to estimate the “utilization distribution” of the scatterplot by microsatellites from each group; the contour containing 92% of the distribution, smoothed using the least-square cross-validation option, was subsequently plotted. The number of distinct alleles and mean PCR fragment length across individuals were calculated from the PCR fragment length dataset. The mean, minimum, maximum, variance, and range in number of repeats across individuals were calculated from the repeat number dataset. The skewness (γ_1_) of the distribution of repeat numbers was calculated using the repeat number dataset and the *skewness* function (moment method) in the *fBasics* package. As the number of humans in the dataset (5435) was markedly higher than the number of chimpanzees (84), a resampling approach was employed when calculating each quantity in the human data. The mean of each variable was calculated across 1,000 sets of 84 individuals drawn randomly (without replacement) from the 5,435 human individuals in the dataset, and these means were then used in all correlations and comparisons; among chimpanzees, quantities were estimated using all 84 individuals. Pearson’s product moment correlation coefficient *r* and Spearman’s rank correlation coefficient *ρ* were calculated using *cor.test*, and Wilcoxon signed-rank and rank-sum tests using *wilcox.test*; both functions are from the *stats* package. In interspecies comparisons, the ARD of a variable was calculated by taking the average across microsatellites of the division of the value in chimpanzees by the value in humans. For within-species comparisons between different microsatellite groups, the RDM of a variable was calculated by dividing its mean among microsatellites in one group by its mean among microsatellites in the other group.

### Availability of supporting data

The datasets supporting the results of this article are included within the article and its additional files.

## Electronic supplementary material

Additional file 1: Table S1: The primer sequences, extracted reference sequence, and the repeat structure identified within that sequence (demarcated by square brackets in the reference sequence), for each of the 221 microsatellites with probable autosomal genomic targets identified in the chimpanzee genome. (XLSX 52 KB)

Additional file 2: Figure S1: Mosaic plots describing microsatellites with **(A)** one, **(B)** two or **(C)** three separate STR regions. (PDF 501 KB)

Additional file 3: Table S2: Genomic coordinates and sequence alignments of the 182 microsatellites with chimpanzee and human orthologs included in the investigation of interspecies non-STR fragment length differences. (XLSX 58 KB)

Additional file 4: Figure S2: Comparison of non-STR ePCR fragment length differences at D6S2410. (PDF 294 KB)

Additional file 5: **A Zip archive containing the dataset comprised of genotypes given as repeat numbers.** (ZIP 1 MB)

Additional file 6: Figure S3: Comparison of pairwise *D*_PS_ calculated on the basis of PCR fragment lengths and repeat numbers. (PDF 1 MB)

Additional file 7: Table S3: The value of each sequence and variability measure investigated for each of the 138 microsatellites included in the *H*_*e*_ correlation analyses and interspecies comparisons. (XLSX 52 KB)

Additional file 8: Table S4: Summary of the properties of the measures of variation across individuals at the 138 microsatellites included in the *H*_*e*_ correlation analyses and interspecies comparisons. (PDF 111 KB)

Additional file 9: Table S5: Spearman’s rank correlations between measures of variation across individuals at the 138 microsatellites included in the *H*_*e*_ correlation analyses and interspecies comparisons. (PDF 267 KB)

## Abbreviations

AMH: Anatomically modern humans
ARD: Average relative difference
*D*_PS_: allele-sharing distance
ePCR: *in silico* PCR
*F*_ST_: Fixation index
*H*_e_: expected heterozygosity
*N*_*e*_: effective population size
PCR: Polymerase chain reaction
RDM: Relative difference in means
*ROS*: Range overlap score
SD: Standard deviation
SNP: Single nucleotide polymorphism
STR: Short tandem repeats.
