# Supplementary material for: Sequence differences at orthologous microsatellites inflate estimates of human-chimpanzee differentiation
Source: BMC Genomics. 2014 Nov 18;15:990. doi: 10.1186/1471-2164-15-990 (PMC4253012; doi:10.1186/1471-2164-15-990)
Supplement: Supplementary file 2 — Additional file 2: Figure S1: Mosaic plots describing microsatellites with (A) one, (B) two or (C) three separate STR regions. (PDF 501 KB) [file 12864_2014_6702_MOESM2_ESM.pdf]

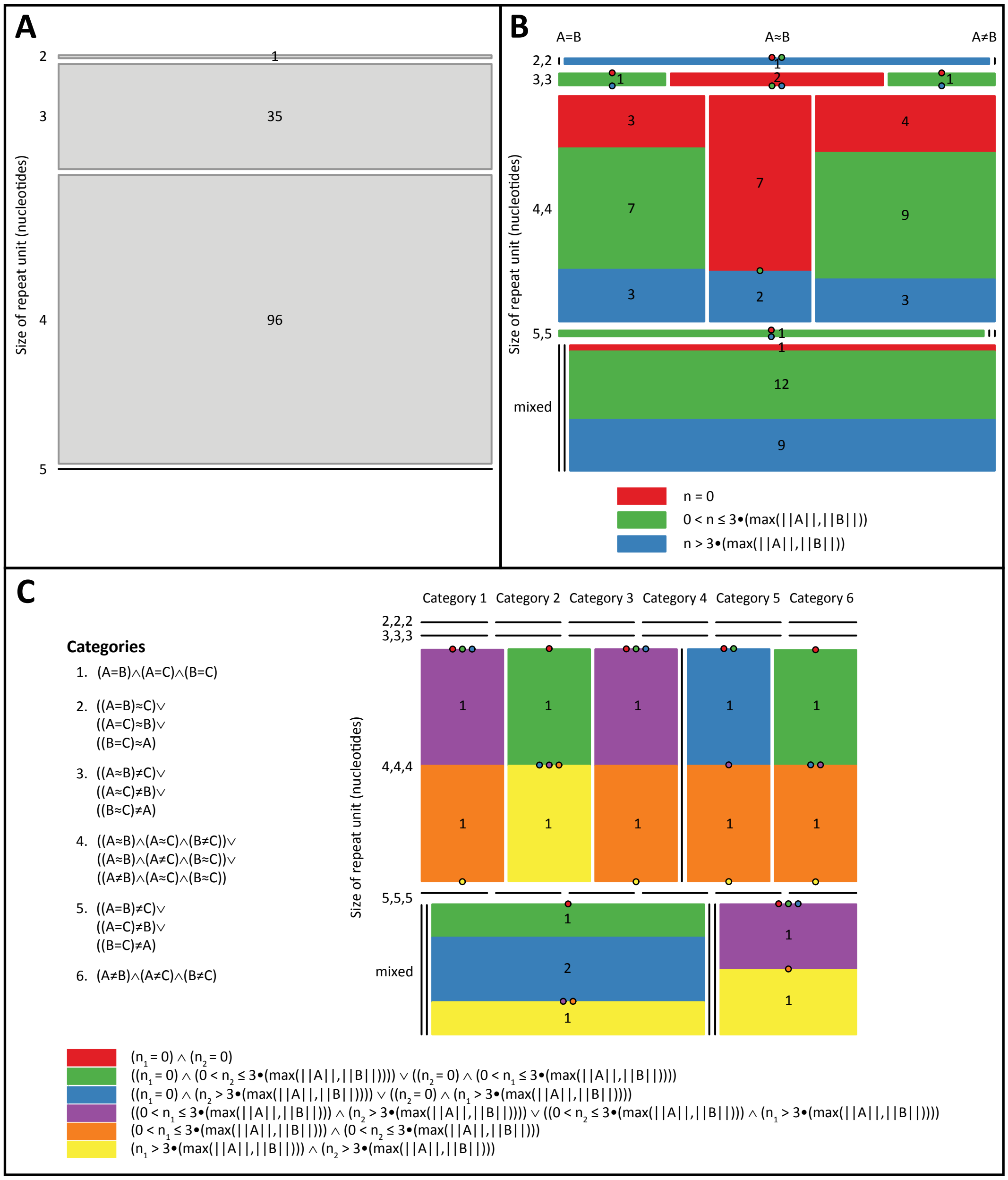

Figure S1. Mosaic plots describing microsatellites with (A) one, (B) two or (C) three separate STR regions. In the mosaic plots [126, 127], tiles represent categories of microsatellites, and the area of each tile is proportional to the number of microsatellites in the corresponding category. For microsatellites with two or more STR regions, microsatellites are grouped by the relationships between the repeat units in their STR regions—identical ("*A=B*"), similar ("*A≈B*"; 1 bp difference between their sequences), or different by more than 1 bp ("*A≠B*")—and by the sizes of those repeat units (i.e. di-, tri-, tetra-, or penta-nucleotide, with "mixed" referring to microsatellites whose STR regions are comprised of repeat units of different sizes). Each group of microsatellites is partitioned into distinct categories based on the distance (in nucleotides) separating their STR regions, as described below the plot, and each category is represented in a different color. Black bars represent groups that contain no microsatellites. Filled circles represent those categories within a group that contain no microsatellites. For microsatellites with two STR regions, n represents the number of nucleotides separating the first and second STR regions. For microsatellites with three STR regions, n_1 and n_2 respectively represent the numbers of nucleotides separating the first & second, and the second & third, STR regions. Key: ∧, and; ∨, or; $\|A\|$, length of A.
