# Supplementary material for: Sequence differences at orthologous microsatellites inflate estimates of human-chimpanzee differentiation
Source: BMC Genomics. 2014 Nov 18;15:990. doi: 10.1186/1471-2164-15-990 (PMC4253012; doi:10.1186/1471-2164-15-990)
Supplement: Supplementary file 4 — Additional file 4: Figure S2: Comparison of non-STR ePCR fragment length differences at D6S2410. (PDF 294 KB) [file 12864_2014_6702_MOESM4_ESM.pdf]

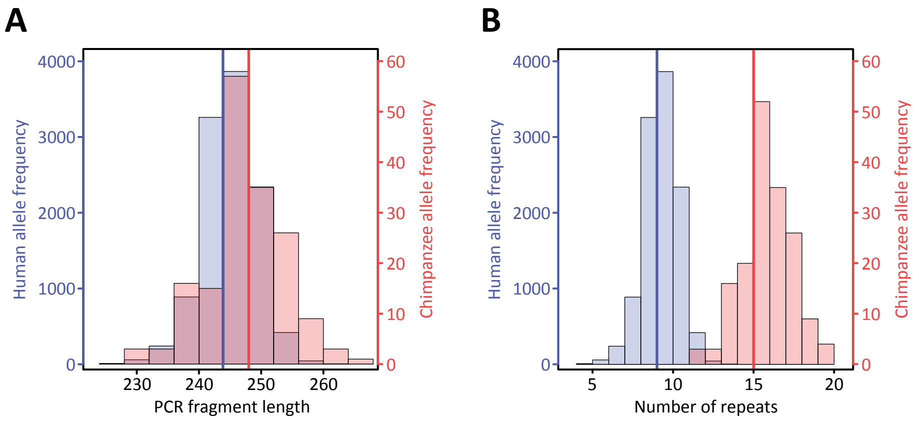

Figure S2. Comparison of non-STR ePCR fragment length differences at D6S2410. For microsatellite D6S2410 the distribution of alleles in humans (blue) and in chimpanzees (red) are shown for (**A**) PCR fragment lengths and (**B**) repeat numbers. The blue and red vertical lines indicate the mean allele size in humans and chimpanzees, respectively.
