# Supplementary material for: Sequence differences at orthologous microsatellites inflate estimates of human-chimpanzee differentiation
Source: BMC Genomics. 2014 Nov 18;15:990. doi: 10.1186/1471-2164-15-990 (PMC4253012; doi:10.1186/1471-2164-15-990)
Supplement: Supplementary file 6 — Additional file 6: Figure S3: Comparison of pairwise D PS calculated on the basis of PCR fragment lengths and repeat numbers. (PDF 1 MB) [file 12864_2014_6702_MOESM6_ESM.pdf]

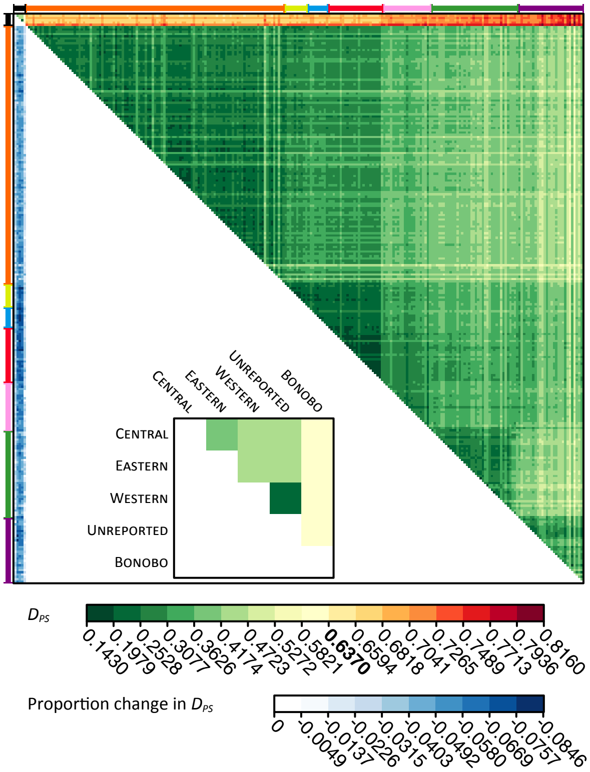

Figure S3. Comparison of pairwise D_{PS} calculated on the basis of PCR fragment lengths and repeat numbers. A heatmap of pairwise D_{PS} values among the 243 non-admixed human and five chimpanzee populations with a sample size of at least five individuals in the human-chimpanzee dataset (upper triangle) and the proportion change in D_{PS} observed when values are calculated using repeat numbers instead of PCR fragment lengths (lower triangle; R^2=0.998). The figure follows the same format as **Figure 5**. Across interspecies comparisons, D_{PS} values calculated using repeat numbers were significantly lower than those calculated using PCR fragment lengths ($P<10^{-16}$, Wilcoxon signed rank test) with a maximum decrease of 8.46% (mean=4.37%, SD=1.53%). Similar reductions are observed when D_{PS} values calculated using repeat numbers at the 138 microsatellites are compared with those calculated on the basis of PCR fragment lengths at all 246 microsatellites in the combined human-chimpanzee dataset (max=8.32%, mean=5.13%, SD=1.17%). The magnitude of the proportion change in D_{PS} when values are calculated using repeat numbers instead of PCR fragment lengths increased with the distance of the human population from Africa (ρ=0.298, $P=2.24\times10^{-6}$).
