# Supplementary material for: Sequence differences at orthologous microsatellites inflate estimates of human-chimpanzee differentiation
Source: BMC Genomics. 2014 Nov 18;15:990. doi: 10.1186/1471-2164-15-990 (PMC4253012; doi:10.1186/1471-2164-15-990)
Supplement: Supplementary file 8 — Additional file 8: Table S4: Summary of the properties of the measures of variation across individuals at the 138 microsatellites included in the H e correlation analyses and interspecies comparisons. (PDF 111 KB) [file 12864_2014_6702_MOESM8_ESM.pdf]

Table S4. Summary of the properties of the measures of variation across individuals at the 138 microsatellites included in the H_e correlation analyses and interspecies comparisons.

**Variable**		**1 STR Region**				**2 STR regions**	
		**Tri** *n*=31		**Tetra** *n*=79		**Tetra** *n*=21	
		**Chimpanzee**	**Human**	**Chimpanzee**	**Human**	**Chimpanzee**	**Human**
Non-STR human-chimpanzee ePCR fragment length differences	Mean	1.81	0.97	3.04	3.66	7.05	7.57
	Minimum	0	0	0	0	0	0
	Maximum	12	10	28	32	26	27
Heterozygosity	Mean	0.58	0.72	0.66	0.72	0.69	0.74
	Minimum	0.05	0.62	0.18	0.47	0.24	0.63
	Maximum	0.87	0.80	0.98	0.86	0.88	0.82
Number of distinct alleles	Mean	8.65	9.41	9.94	8.08	13.24	8.89
	Minimum	2	6.98	2	5.44	6	6.03
	Maximum	18	18.06	29	16.08	27	12.92
Variance in the number of repeats	Mean	2.84	3.92	3.26	2.44	4.48	2.61
	Minimum	0.09	1.81	0.14	0.61	1.00	0.78
	Maximum	9.35	9.85	17.36	11.89	14.28	6.76
Range of the number of repeats	Mean	8.40	9.41	9.39	8.27	11.25	9.05
	Minimum	2	7.09	2	5.46	6	6.12
	Maximum	15	12.55	19	19	17.25	18.34
Mean PCR fragment length	Mean	182.67	189.97	187.91	194.26	193.36	202.83
	Minimum	114.31	112.61	89.83	107.48	114.90	137.13
	Maximum	269.71	288.38	313.81	319.27	264.84	281.61
Mean number of repeats	Mean	10.27	13.22	9.99	11.58	14.10	16.66
	Minimum	4.96	9.62	3.68	5.33	8.09	13.39
	Maximum	15.15	17.70	15.18	16.02	17.99	22.07
Maximum number of repeats	Mean	14.51	17.58	14.45	15.05	19.32	20.43
	Minimum	8	14.02	4	8.29	12	16.63
	Maximum	20	23.33	27	25	25	26.30
Minimum number of repeats	Mean	7.11	9.17	6.06	7.79	9.07	12.39
	Minimum	3	6.82	1	0.92	1	8.96
	Maximum	11	11.94	12	12.25	13.25	17.88

Microsatellites were grouped by the number of separate STR regions and their repeat unit size. No values are presented for microsatellites with one dinucleotide STR region, two trinucleotide STR regions, or three or four tetranucleotide STR regions because of small sample sizes (1, 1, 4, and 1, respectively).
