# Supplementary material for: Sequence differences at orthologous microsatellites inflate estimates of human-chimpanzee differentiation
Source: BMC Genomics. 2014 Nov 18;15:990. doi: 10.1186/1471-2164-15-990 (PMC4253012; doi:10.1186/1471-2164-15-990)
Supplement: Supplementary file 9 — Additional file 9: Table S5: Spearman’s rank correlations between measures of variation across individuals at the 138 microsatellites included in the H e correlation analyses and interspecies comparisons. (PDF 267 KB) [file 12864_2014_6702_MOESM9_ESM.pdf]

Table S5. Spearman's rank correlations between measures of variation across individuals at the 138 microsatellites included in the H_e correlation analyses and interspecies comparisons.

Variable			Number of distinct alleles		Variance in the number of repeats		Range of the number of repeats		Mean PCR fragment size		Mean number of repeats		Maximum number of repeats		Minimum number of repeats	
			Chimp	Human	Chimp	Human	Chimp	Human	Chimp	Human	Chimp	Human	Chimp	Human	Chimp	Human
Number of distinct alleles	Tri	ρ	—	—	**0.610**	**0.515**	**0.706**	**0.823**	0.219	0.050	**0.657**	0.189	**0.690**	**0.397**	0.275	-0.072
		P	—	—	**2.73×10^{-4}**	**0.003**	**9.24×10^{-6}**	**7.42×10^{-7}**	0.237	0.787	**5.99×10^{-5}**	0.307	**1.78×10^{-5}**	**0.028**	0.135	0.700
	Tetra	ρ	—	—	**0.571**	**0.698**	**0.682**	**0.904**	0.028	0.113	**0.232**	**0.294**	**0.432**	**0.528**	-0.088	0.046
		P	—	—	**3.87×10^{-8}**	**8.70×10^{-13}**	**4.62×10^{-12}**	**$<10^{-16}$**	0.806	0.321	**0.040**	**0.009**	**6.93×10^{-5}**	**5.80×10^{-7}**	0.438	0.689
Variance in the number of repeats	Tri	ρ	—	—	—	—	**0.710**	**0.581**	0.010	-0.194	**0.454**	-0.202	**0.471**	0.061	-0.091	-0.274
		P	—	—	—	—	**7.78×10^{-6}**	**0.001**	0.958	0.294	**0.011**	0.274	**0.008**	0.743	0.627	0.136
	Tetra	ρ	0.298	**0.835**	—	—	**0.569**	**0.738**	0.103	0.020	-0.036	-0.078	0.169	0.158	**-0.293**	**-0.265**
		P	0.190	**5.13×10^{-8}**	—	—	**4.42×10^{-8}**	**8.89×10^{-15}**	0.367	0.864	0.752	0.494	0.137	0.164	**0.009**	**0.018**
Range of the number of repeats	Tri	ρ	—	—	—	—	—	—	0.006	-0.125	**0.511**	0.231	**0.795**	**0.490**	0.079	-0.053
		P	—	—	—	—	—	—	0.975	0.500	**0.003**	0.211	**9.45×10^{-8}**	**0.006**	0.673	0.777
	Tetra	ρ	**0.469**	**0.864**	**0.689**	**0.843**	—	—	0.123	0.133	**0.275**	**0.283**	**0.559**	**0.515**	-0.191	-0.042
		P	**0.032**	**$<10^{-16}$**	**5.46×10^{-4}**	**$<10^{-16}$**	—	—	0.280	0.242	**0.014**	**0.011**	**8.39×10^{-8}**	**1.22×10^{-6}**	0.091	0.712
Mean PCR fragment size	Tri	ρ	—	—	—	—	—	—	—	—	-0.003	0.098	0.113	0.067	0.106	0.152
		P	—	—	—	—	—	—	—	—	0.989	0.600	0.544	0.721	0.571	0.413
	Tetra	ρ	0.084	0.143	-0.027	0.066	0.265	0.162	—	—	0.151	0.028	0.103	0.031	0.040	0.020
		P	0.717	0.535	0.908	0.776	0.245	0.480	—	—	0.184	0.805	0.367	0.784	0.727	0.860
Mean number of repeats	Tri	ρ	—	—	—	—	—	—	—	—	—	—	**0.776**	**0.896**	**0.714**	**0.875**
		P	—	—	—	—	—	—	—	—	—	—	**2.84×10^{-7}**	**1.60×10^{-7}**	**6.38E-06**	**3.05E-07**
	Tetra	ρ	-0.031	0.370	0.043	0.357	-0.032	**0.566**	0.013	0.069	—	—	**0.857**	**0.915**	**0.747**	**0.864**
		P	0.894	0.099	0.854	0.113	0.891	**0.008**	0.957	0.767	—	—	**6.53×10^{-24}**	**0**	**2.56E-15**	**0**
Maximum number of repeats	Tri	ρ	—	—	—	—	—	—	—	—	—	—	—	—	**0.603**	**0.822**
		P	—	—	—	—	—	—	—	—	—	—	—	—	**3.27E-04**	**7.50E-07**
	Tetra	ρ	0.224	**0.486**	**0.561**	**0.483**	**0.565**	**0.684**	-0.043	-0.027	**0.690**	**0.970**	—	—	**0.644**	**0.763**
		P	0.328	**0.027**	**0.008**	**0.028**	**0.008**	**8.59×10^{-4}**	0.853	0.908	**5.39×10^{-4}**	**4.94×10^{-6}**	—	—	**1.50E-10**	**0**
Minimum number of repeats	Tri	ρ	—	—	—	—	—	—	—	—	—	—	—	—	—	—
		P	—	—	—	—	—	—	—	—	—	—	—	—	—	—
	Tetra	ρ	-0.312	-0.312	-0.179	-0.296	-0.429	-0.199	-0.256	-0.255	**0.792**	**0.500**	0.416	0.406	—	—
		P	0.168	0.169	0.437	0.192	0.052	0.386	0.263	0.264	**1.89×10^{-5}**	**0.022**	0.061	0.069	—	—

Spearman's rank correlation coefficients (ρ) and their associated P values are shown for comparisons of the measures of variation across individuals in the dataset for microsatellites with one (upper triangle) or two (lower triangle) STR regions embedded in their sequence. Microsatellites were grouped by their number of separate STR regions and repeat unit size. Sample sizes for loci with one STR region were 31 and 79 for tri- and tetra-nucleotides, respectively, and for loci with two separate STR regions they were 0 and 21, respectively. Correlations with $P<0.05$ are shown in **bold**.
